# Photocatalyst size controls electron and energy transfer: selectable *E*/*Z* isomer synthesis *via* C–F alkenylation†
†Electronic supplementary information (ESI) available. See DOI: 10.1039/c6sc02422j



**DOI:** 10.1039/c6sc02422j

**Published:** 2016-07-21

**Authors:** A. Singh, C. J. Fennell, J. D. Weaver

**Affiliations:** a Department of Chemistry , Oklahoma State University , Stillwater , OK 74078 , USA . Email: jimmie.weaver@okstate.edu

## Abstract

Photocatalytic alkene synthesis can involve electron and energy transfer processes. The structure of the photocatalyst can be used to control the rate of the energy transfer, providing a mechanistic handle over the two processes. Jointly considering catalyst volume and emissive energy provides a highly sensitive strategy for predicting which mechanistic pathway will dominate. This model was developed *en route* to a photocatalytic C_aryl_–F alkenylation reaction of alkynes and highly-fluorinated arenes as partners. By judicious choice of photocatalyst, access to *E*- or *Z*-olefins was accomplished, even in the case of synthetically challenging trisubstituted alkenes. The generality and transferability of this model was tested by evaluating established photocatalytic reactions, resulting in shortened reaction times and access to complimentary *Z*-cinnamylamines in the photocatalytic [2 + 2] and C–H vinylation of amines, respectively. These results show that taking into account the size of the photocatalyst provides predictive ability and control in photochemical quenching events.

## Abstract 2

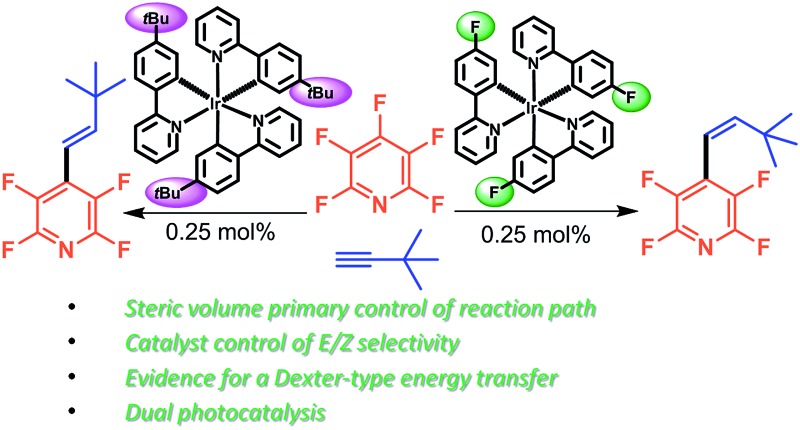
This work highlights the role of photocatalyst volume in controlling photoquenching events and is exploited to control the reaction outcomes.

## Introduction

In recent years, the number of new photocatalytic methods has grown rapidly.^1^ A key step in the vast majority of these methods is an electron transfer to or from an excited state photocatalyst and the substrate, reductant or oxidant. Under the right circumstances, however, the excited state of the same photocatalysts can undergo energy transfer to the substrate rather than electron transfer.^2^ There are even fewer examples in which both electron- and energy-transfer both take place in the same reaction. One potential reason for this is that the factors that dictate these two fundamentally different processes are underexplored, and it is not well understood what causes one process to be dominant when both are possible. A clearer understanding of such factors would provide valuable insight for the further development of the field of photocatalysis.

For two reasons, we envisioned that the photocatalytic synthesis of alkenylated fluoroarenes *via* the formal hydrofluoroarylation of alkynes from perfluoroarenes would provide an ideal platform for exploring how the nature of the photocatalyst influences these processes (Fig. 1a). First, we have shown that the C–F bond of multifluorinated arenes can be functionalized *via* photocatalytic electron transfer.^3^ Secondly, we^4^ and others^5^ have shown that the same photocatalyst can engage in selective energy transfer, leading to styrenes enriched in the thermodynamically less favorable *Z*-isomer.^4^ We performed gas-phase geometry optimizations of ***E*-1a** and ***Z*-1a** which revealed a significant difference in the dihedral angle between the pyridine ring and the double bond (Fig. 1b). In the B3LYP 6-31G* optimized structures shown, the steric clash between *o*-arene fluorines and the alkene chain in ***Z*-1a** significantly increases the dihedral angle and hinders extended conjugation. In our experience, the difference in dihedral angle has been a good indicator of the ability to achieve good photostationary state selectivity (*i.e.* high *Z* : *E* ratios).^4^

**Fig. 1:**
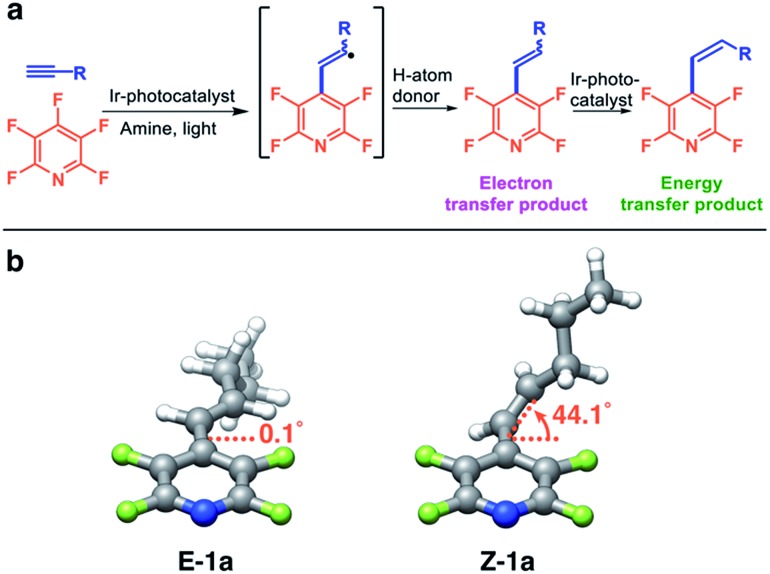
(a) The proposed reaction scheme. (b) Optimized gas-phase geometries for ***E*-1a** and ***Z*-1a** from DFT calculations and the dihedral angle between alkene and arene, leading to differences in conjugation and photostationary states.

Consequently, we expected that the styrenyl-like product of electron transfer would also be a competent quencher of the excited state photocatalyst. Control of the rates of these fundamental photoquenching processes would be essential to both forming the product and controlling the double bond geometry.

A survey of the literature for processes that might involve both electron and energy transfer provides some insight into how to control the olefin geometry. For instance, MacMillan showed that the rate of the isomerization of cinnamyl amines could be substantially reduced by changing from dimethylacetamide (DMA) to toluene as the solvent (eqn (1), Scheme 1).^6^ Alternatively, Qing^5*c*^ showed that electron rich styrenes could undergo an oxidative β-trifluoromethylation and isomerization (eqn (2)). In this case, the thermodynamic *E*-isomer is likely also the kinetic product. Consequently, the *E*/*Z* selectivity could be controlled by the choice of photocatalyst. Use of Ru(bpy)_3_Cl_2_·6H_2_O whose excited state emissive energy is 46.5 kcal mol (ref. 1*a*) makes the energy transfer process substantially endergonic (53.2 kcal mol^–1^ for *trans*-β-methylstyrene)^7^ and sufficiently slow that the isolation of the kinetic product is possible. In contrast, use of Ir(ppy)_3_ whose emissive energy is 55.2 kcal mol^–1^ (ref. 8) is of sufficient triplet state energy to isomerize the *trans*-styrenyl product selectively leading to an enrichment of the *Z*-isomer at the photostationary state.

**Scheme 1:**
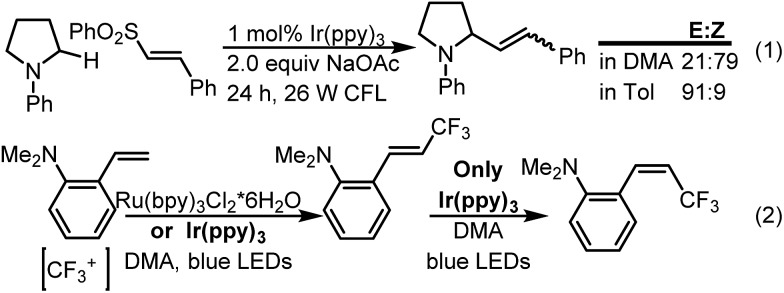
Strategies used to control energy transfer.

For our proposed reaction system, both electron and energy transfer occur in the same solvent. Furthermore, the available photocatalysts which are sufficiently reducing to enable the C–F functionalization are also sufficiently energetic to facilitate the isomerization. Thus, we could not rely on either a solvent switch or the emissive energy of the photocatalyst to provide the needed control as has been done previously. A less explored facet of the energy transfer process in photocatalysis is the efficiency of energy transfer as a function of internuclear distance. Both the Förster^9^ and the Dexter^10^ energy transfer mechanisms show significant distant dependency in the rate of energy transfer, suggesting that the steric volume of the photocatalyst could potentially be used as a design element to turn on or off energy transfer despite its emissive energy.

## Results and discussion

We began our investigation with conditions that had previously facilitated photocatalytic C–F functionalization.^3^ Our first objective was to find conditions that allowed the C–C bond formation, regardless of the olefin geometry, rather than the hydrodefluorinated product^3*a*^ (**2b**). In a solvent screen, MeCN proved to be the superior solvent with DMSO a close second (Table 1, entries 1–5). Next, a screening of the alkyne loading showed a direct correlation between the concentration of alkyne and the relative amount of product, **2a**, formed (entries 1 and 6–8) with 6 equivalents being optimal. While the reaction was not particularly sensitive to air (entry 9), it did require light, amine, and photocatalyst (entry 10). Finally, we lowered the temperature and examined the effect of amine concentration (entries 11–14).^11^ Decreasing the amount of amine gave a better **2a**/**2b** ratio but at the cost of conversion. However, the decreased yield could be overcome by adding the amine incrementally over time (entry 14) which resulted in 84% conversion to the product. In all cases the observed *Z* : *E* ratio never exceeded 1.3 : 1.

**Table 1:** Optimization of reaction conditions

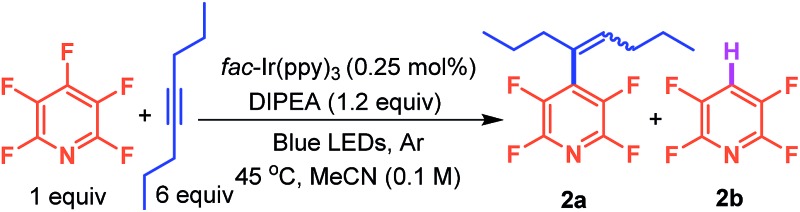				
Entry	Modifications	**2a**/**2b**	Conv. to **2a** ^*a*^ ^,^ ^*c*^	Time, h
1	None	4.9	65	17
2	THF, DCM, ether, toluene instead of MeCN	na	na	17
3	Acetone instead of MeCN	3.8	12	17
4	DMF, DMA, NMP instead of MeCN	<0.6	<28	17^*b*^
5	DMSO instead of MeCN	3.6	61	17^*b*^
6	1.2 equiv. 4-octyne	1.5	44	15
7	2.0 equiv. 4-octyne	2.2	29	15
8	4.0 equiv. 4-octyne	4.0	44	15
9	w/o degassing	4.7	61	18
10	Dark, no DIPEA, or no Ir(ppy)_3_	na	na	<14
11	At 0 °C with 0.25 equiv. DIPEA	8.7	30/36	15/35
12	At 0 °C with 1.0 equiv. DIPEA	6.0	41/72	15/35
13	At 0 °C with 2.0 equiv. DIPEA	3.9	55/70	15/35^*b*^
14	At 0 °C with incremental addition of DIPEA 0.5–0.75 equiv.	9.2	57/84	17/37

^*a*^Determined by ^19^F NMR.

^*b*^Full conversion.

^*c*^ *Z*/*E* selectivity < 1.3.

Using the optimal conditions found in Table 1 (entry 14) we sought to evaluate the effect of the catalyst on the *E* : *Z* selectivity. We chose *t*-butylacetylene because it was expected to have a strong kinetic preference for the *E*-isomer (Fig. 2a),^12^ which would allow us to determine whether isomerization occurs. Additionally, we postulated that the steric bulkiness of the *t*-butyl group would make the substrate more sensitive to changes in volume of the photocatalyst. Using our library of photocatalysts^8^ which met two of three criteria, we evaluated the ability to facilitate the electron transfer and isomerization. The criteria included a demonstrated ability in C–F functionalization, had a reduction potential of –1.5 V (*vs.* SCE) or more negative from either their excited state or reduced ground state, and an emissive energy that was at least 51 kcal mol^–1^. While the conversions varied depending on which catalyst was used,^12^ the ratio of the isomers at the photostationary state were recorded.

**Fig. 2:**
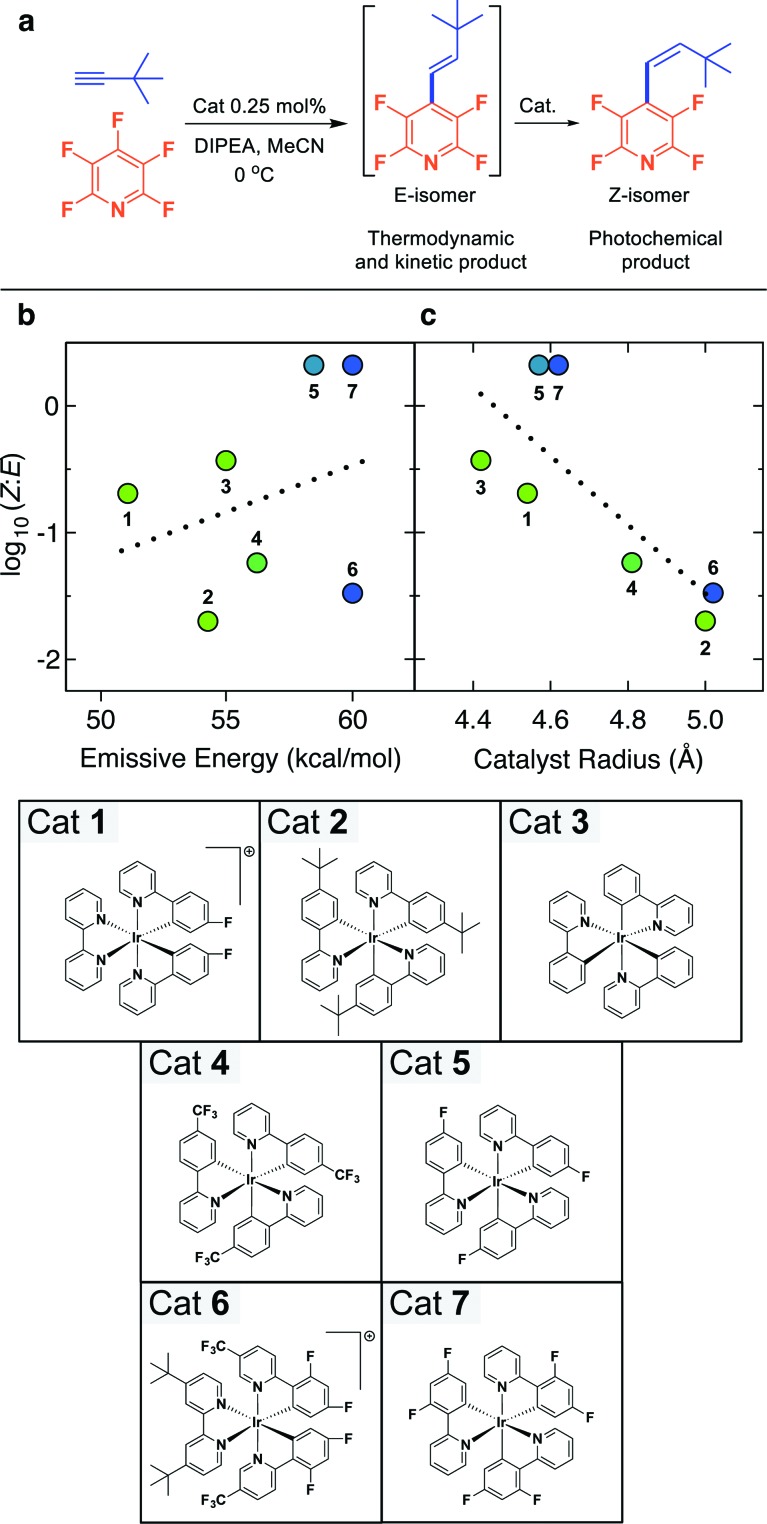
(a) The reaction scheme for the photocatalyst selectivity investigation, (b) scatter plot of the log(*Z* : *E*) as a function of the emissive energy of the labeled photocatalysts which were taken from the literature and correspond to the emission spectrum *λ*_max_,^8^ and (c) scatter plot of the log(*Z* : *E*) as a function of the effective radius of labeled photocatalysts, colored by the measured emissive energy. Conversion to the strained *Z*-isomer is increasingly less effective with increasing catalyst size, though catalysts with high emissive energies can deviate from this trend. Cationic catalysts are PF_6_^–^ salts.

A plot of emission energy of the photocatalyst *vs.* the log *Z* : *E* ratio showed no correlation (*R* = 0.30, Fig. 2b). For instance, the catalyst with the lowest emissive energy (**Cat 1**) gives the middle log *Z* : *E* value, while increases in emissive energy of the catalyst lead to both increased and decreased log *Z* : *E* values. This is somewhat surprising given classic studies involving the sensitized isomerization of stilbenes typically displayed a strong correlation with the energy of the sensitizer.^13^ However, in contrast to many of the classic sensitizers; benzophenone, xanthone, *etc.* which tend to be planar, structurally 2-dimensional in nature, the ligation of iridium with three bidentate ligands results in a 3-dimensional molecule that is somewhat spherical. Therefore, it stood to reason that the sterics of the catalyst might be an important factor.

In order to assess the role of photocatalyst size, we performed TPSS density functional theory geometry optimizations of all the tested photocatalysts using the QZVP basis set,^14^ an accurate combination for treating noble metal complexes.^15^ We found the calculated geometry of *fac*-Ir(ppy)_3_ (*fac*-tris[2-phenylpyridinato-*C* ^2^,*N*]iridium(iii)) to be in good agreement with a known gas-phase electron diffraction (GED) structure and we expect a similar level of agreement to future GED structures for the other investigated photocatalysts.^12,16^ The volume of each photocatalyst was determined from numerical integration of the 0.001 electrons per Å^3^ electron density isosurface, and we converted these volumes to an effective catalyst radius for simplicity by assuming a spherical shape. The plot of the log *Z* : *E* as a function of this radius, generally, shows a linear trend (*R* = –0.78, Fig. 2c). As the steric volume of the photocatalyst increases, the propensity to undergo isomerization decreases. Deviations that favor conversion to the *Z*-isomer occur when high emissive energy photocatalysts are used. These deviations appear to correlate with the difference between the emissive energy of the photocatalyst and the perpendicular triplet state molecule (*i.e. E*_tp_ of 53 kcal mol^–1^) which has undergone 90° rotation about the former double bond.^7^ Higher emissive energy of a catalyst, such as that seen with Ir(diFPhCF_3_Pyr)_2_dtbbpy^+^ (**Cat 6**) (60.1 kcal mol^–1^), can work to counter their bulk compared to also bulky **Cat 2**. Likewise, catalysts can be relatively inefficient at isomerization even though the steric volume is very small, such as in the case of Ir(Fppy)_2_bpy^+^ (**Cat 1**) where the energy transfer is endergonic. It should be noted that the increase in steric volume is coupled with an increase in degrees of freedom and corresponding modes for energy relaxation, and this may also play a role in the decrease in efficiency.

We next investigated whether this trend of selectivity as a function steric bulk could be used to control reactions across a wider variety of substrates. The extreme isomerization efficiency of *fac*-Ir(diFppy)_3_ (**Cat 7**) resulted in product inhibition, making it a poor catalyst for the investigated C–F functionalization. However, by using photocatalysts with small volumes, such as *fac*-Ir(ppy)_3_ (**Cat 3**) or *fac*-Ir(Fppy)_3_ (**Cat 5**), we were able to directly obtain the endergonic, less conjugated (usually *Z*-isomer) alkenylated product. Indeed, we observed that a variety of perfluoroarenes and alkynes could be coupled and isomerized in good yields and high selectivity (Table 2a). The standard conditions worked well to make a range of products, including sterically bulky products (**3a–5a**, **10a–11a**, and **21a**) and those with an adjacent methine (**8a**, **9a**, and **12a**). These conditions also worked for some terminal alkynes that gave allylic methylenes (**15a**), but for some substrates we found that the isomerization was slow in MeCN. By simply performing a solvent switch to DMF the desired isomer was obtained in high selectivity (**11a**, **14a**, **16a**, and **17a**).^17^ Single isomers of trisubstituted alkenes could be obtained by photocatalytic addition/isomerization of internal alkynes (**6a**, **13a**, and **14a**). While the method proved regioselective with respect to the alkyne in the case of the electronically unsymmetric alkynes (**6a**), it was only marginally regioselective in the case of sterically unsymmetric internal alkynes.^18^ In general, selective production of the *Z*-isomer by using small photocatalysts appears be most useful for terminal, symmetric internal, or electronically differentiated internal alkynes. Electron rich ethoxyacetylene also underwent smooth addition, but resulted in a mixture of *E*/*Z* isomers (**18a**), potentially a result of a lowered triplet state energy or a decreased HOMO–LUMO gap. Nonetheless, if desired, both isomers can serve as a surrogate of an aldehyde *via* hydrolysis. Both **20a** and **21a** indicate the preference for fragmentation of C–Cl and C–Br over the C–F and illustrate how it can be used in a complimentary fashion to access alternative regioisomers. Current methods for vinylated multifluorinated arenes are generally more circuitous. The direct photocatalytic alkenylation of the C–F bond shortens the sequence necessary to access this motif and significantly increases the accessible chemical space.

**Table 2:** Photocatalytic C–F alkenylation/isomerization

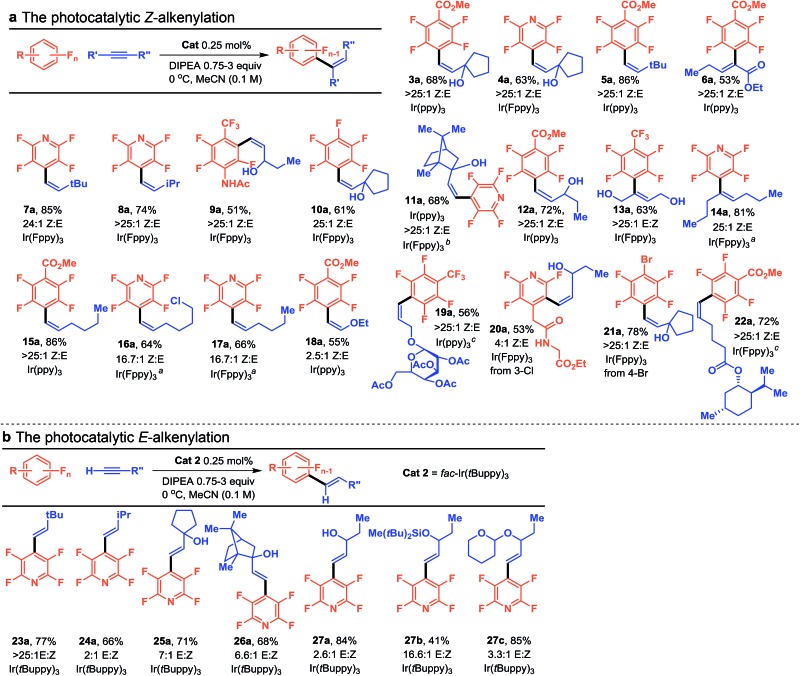

^*a*^The volatiles were removed and DMF was added and the reaction irradiated.

^*b*^The product was chromatographed to remove original catalyst then resubjected to isomerization with new catalyst and DMF.

^*c*^Alkyne was limiting reagent.

Under these conditions, in the highly fluorinated pyridine system, the C_2_–F is also labile under electron transfer conditions.^3*b*^ However, because of the increased propensity of the photocatalyst towards energy transfer which we used and the presence of the alkenylated product the energy transfer becomes the dominant pathway and can serve to protect functional groups sensitive to photochemical electron transfers (*i.e.* over reduction). Product **21a** serves as a dramatic example of this feature. With this product, no hydrodebromination is observed despite the sensitivity of C–Br to further reduction. This likely occurs because energy transfer to the alkene effectively outcompetes electron transfer needed for C–Br rupture. Finally, we have shown (**19a** and **22a**) that the alkyne can be used as the limiting reagent, albeit at the expense of excess perfluoroarene. It is expected that this will be useful in the case that the alkyne is the valuable component, giving an added versatility to the method.

Next, we turned our attention to performing the coupling without subsequent isomerization (Table 2b). We expected that by utilizing *fac*-Ir(*t*Buppy)_3_ (**Cat 2**), which has a relatively large volume and moderate emissive energy, the kinetic alkenylation product (*E*-isomer typically) should be selectively favored since isomerization would be slow. Indeed, for sterically larger alkynes, the selectivity ranges from excellent (**23a**) to good (**25a** and **26a**), giving the *trans*-isomer as the major product. Less sterically demanding substrates show significantly diminished selectivities (**24a** and **27a**), though it may be possible that the selectivity could be increased by considering H-atom sources with even greater steric bulk or lowering the temperature. Finally, given the importance of allylic alcohols in synthesis, we evaluated the effect of common protecting groups on the selectivity. While the tetrahydropyran protected alcohol (**27c**) resulted in only modest improvement over the unprotected alcohol, the bulky silyl protecting group (**27b**) resulted in excellent improvement to selectivity 16.1 : 1 E : Z. Thus, by simply switching to a highly reducing, but sterically large photocatalyst we can access the complimentary kinetic product.

Finally, having demonstrated catalyst control over product geometry in the photochemical C–F alkenylation, we wanted to probe the generality of our understanding towards other photocatalyzed processes. First, we examined Yoon's [2 + 2] cycloaddition reaction in which he demonstrated that energy transfer from **Cat 6** to non-polarized styrenes could yield cyclobutanes.^2*a*^ When we performed the reaction using several photocatalyst of varying volumes and emissive energies, we observed the expected trend for all the substrates we checked. The smaller catalysts in these cases may also benefit from faster diffusion, resulting in increased conversion rates. Additionally, we saw that the sensitivity increased with the steric demand of the substrate (Table 3). Though we did not include Ir(CF_3_dFppy)_2_bpy^+^ in our initial screen, which is similar in emissive energy to **Cat 6** but sterically less demanding (*i.e.* missing the *t*Bu groups), it outpaced catalyst (**Cat 6**) used by Yoon.

**Table 3:** Photocatalyzed [2 + 2] and C–H styrenylation of amines

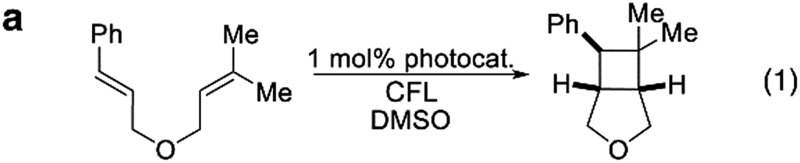			
Photocatalyst	Radius (Å)	Emissive energy (kcal mol^–1^)	Conv.^*a*^ 18 h
**Cat 5**	4.57	58.6	74%
**Cat 7**	4.62	60.1	68%
**Cat 6** ^*b*^	5.02	60.1	61%
**Cat 2**	5.00	54.5	10%

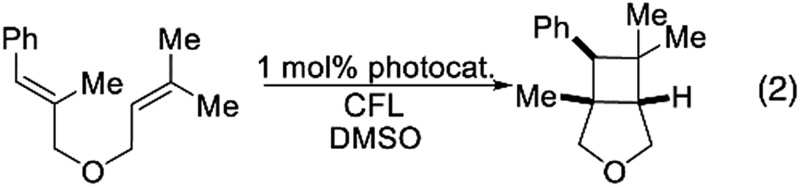			
**Cat 7**	4.62	60.1	63%
**Cat 5**	4.57	58.6	53%
lr(CF_3_dFppy)_3_bpy^+^	4.65	60.1	48%
**Cat 6** ^*b*^	5.02	60.1	35%

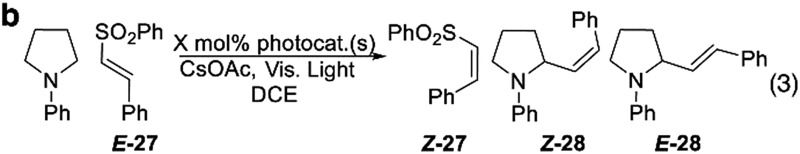					
Entry	Photocatalyst	mol%	*E*-27 : *Z*-27 : *Z*-28 : *E*-28^*c*^	Conv.^*d*^	Time
1	**Cat 6** ^*e*^	1%	7 : 0 : 1 : 92	62%	24.0 h
2	**Cat 7**	1%	25 : 53 : 5 : 16	14%	24.0 h
3	**Cat 2**	1%	0 : 0 : 0 : 100	100%	10.5 h
4	**Cat 7**/**2**	0.5/0.125%	0 : 0 : 91 : 9	100%	18.0 h (94%)^*f*^

^*a*^Determined by ^1^H NMR on reaction mixture after extraction and concentration.

^*b*^Yoon *et al.* conditions.^19^

^*c*^Ratios determined by GCMS.

^*d*^Determined by H NMR.

^*e*^MacMillan *et al.* conditions.^6^

^*f*^Isolated as a 82 : 18 *Z* : *E* mixture.

We next considered MacMillan's styrenylation of aniline derivatives. In this reaction, electron transfer is believed to give rise to the α-amino radical which then undergoes an attack on the vinyl sulfone, followed by fragmentation of the C–S bond to regenerate the double bond. The kinetic product of the elimination is the *E*-alkene. Our findings can also help explain seemingly contradictory results from the literature. Specifically, MacMillan observed^6^ that use of the Ir(CF_3_dFppy)_2_dtbubpy^+^ (**Cat 6**) gave the *E*-product selectively, despite the favorable energetics (60.1 kcal mol^–1^ emissive energy)^1*a*^ for isomerization. However, despite its high energy, we would predict **Cat 6** would only inefficiently isomerize the product and therefore is ideal for electron transfer and thus would afford the *E*-product. After we confirmed MacMillan's result (entry 1, Table 3b), we attempted to directly access the *Z*-isomer. However, we found it was not feasible simply using a photocatalyst more capable of isomerization (**Cat 7**, entry 1). In this case, unproductive isomerization of the starting sulfone, ***E*-27** is the dominant photoquenching pathway and thus an overall inefficient process. Given that isomerization of the starting material (***E*-27** to ***Z*-28**) is an energy wasting method in which **Cat 6** may have engaged unnoticed, it not surprising that **Cat 2** which is very inefficient at isomerization gave the *trans*-product ***E*-28** exclusively at a considerably greater rate than **Cat 6**. Finally, by combining **Cat 7** and **Cat 2** we hoped to accomplish both photochemical processes (entry 4). Indeed, we were able to accomplish both the electron transfer and isomerization processes by using two different photocatalysts simultaneously. The loadings were adjusted to try to balance the rates and ultimately using just 0.5 mol% of **Cat 7** and 0.125 mol% of **Cat 2** we were able to isolate the ***Z*-28** in high yield, 94%, and diastereoselectivity (82 : 18).

Our current understanding is shown in Scheme 2. In which absorption of a photon gives rise to an excited state catalyst, PC*. In the absence of an isomerizable group, PC* may enter the electron transfer cycle. In which oxidative or reductive quenching may be at work depending on photocatalyst and substrate choice.^3*b*^ Ultimately, in the case of perfluoroarenes, electron transfer to the perfluoroarene ArF_*n*_ leads to fluoride extrusion and formation of a multifluorinated aryl radical, ArF_*n*–1_.

**Scheme 2:**
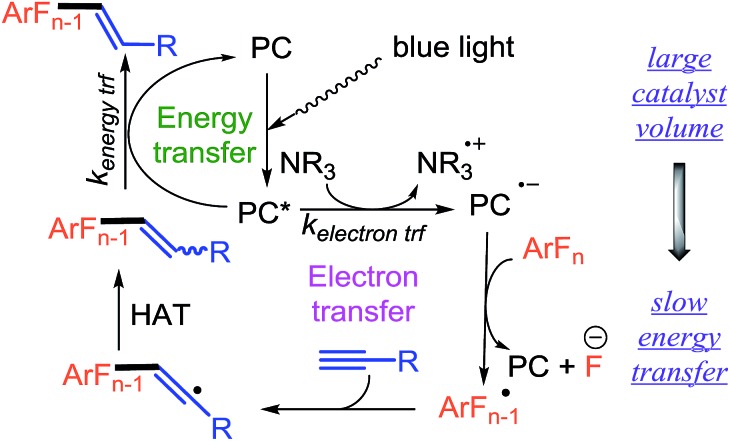
Working mechanism.

This reactive radical will engage the alkyne to form a vinyl radical. The vinyl radical undergoes HAT with selectivity that depends upon the size of the R group to give a new styrenyl-like substrate. The *trans*-styrenyl motif can then engage the photocatalyst in an energy transfer mechanism leading to preferential isomerization to the *cis*-alkene.^4^ Here, we have shown that the rate of isomerization strongly correlates to the catalyst volume and is easily exploited to manipulate the reaction.

## Conclusions

We have observed that the size of a photocatalyst can be used as a sensitive control in determining the rate of energy transfer in photocatalytic C–F alkenylation as well as other photocatalyzed processes. While we are continuing to explore the limits and nature of this control, these findings can also help explain other seemingly contradictory results. For instance, in the styrenylation of amines,^6^ MacMillan observed that use of the Ir(CF_3_dFppy)_2_dtbubpy^+^ photocatalyst did not result in isomerization of the double bond, this despite the favorable energetics (60.1 kcal mol^–1^ emissive energy).^1*a*^ Based on the size of the photocatalyst, this result is expected. Sensitivity to the 3-dimensional structure of the catalyst is a relatively unexplored facet of photocatalysis. Given our observed distance dependence sensitivity, energy transfer from Ir-based photocatalysts, which leads to isomerization seems consistent with a Dexter mechanism in which a simultaneous exchange of electrons demands good orbital overlap. The level of intimacy between the photocatalyst and substrate required for energy transfer to occur suggest an exciting future with further developments exploiting this distance dependence.
